# Supplementary material for: Psychometric Properties of the Italian Version of the 25-Item Hikikomori Questionnaire
Source: Int J Environ Res Public Health. 2022 Oct 19;19(20):13552. doi: 10.3390/ijerph192013552 (PMC9603413; doi:10.3390/ijerph192013552)
Supplement: Supplementary file 1 [file ijerph-19-13552-s001.zip › ijerph-1938386-supplementary.pdf]

Psychometric Properties of the Italian Version of the 25-Item Hikikomori Questionnaire

Power analysis for determining the optimal sample size

As one of main aim of our study was to confirm the three-factor structure of the HQ-25 (Teo et al., 2018), we estimated the optimal sample size given an alpha-level of 0.05 and a power of 0.90, we considered two different CFA models assuming different effect sizes: the first one is the HQ-25 three-factor solution and the second one is the one-factor solution. Specifically, we computed the optimal sample size for detecting a three-factor solution against the one-factor solution given an alpha-level, a power, an a priori pre-specified factor loading matrix and a priori pre-specified latent factor correlations. Two different a priori factor loading matrices were hypothesized: in the first one we considered the factor loading matrix obtained from the exploratory factor analysis of the HQ-25 and published in Table 1 of Teo et al.'s (2018) study. Those factor loadings were on average well above 0.50 on all the three factors. Then, in the second factor loading matrix we hypothesized that all items loaded on the specific factor with a fixed factor loading of 0.50. This pattern should represent a more pessimistic scenario than the pattern of factor loadings by Teo et al. (2018). Further, Teo et al. (2018) did not report factor correlations. Thus, to contrast the three-factor model with the one-factor model, we again hypothesized a pessimistic scenario assuming that in the priori factor model the three factors have a very high latent correlation of 0.8, yet not equal to 1 (in this last case, we should assume that the three factors are the same and the one-factor model is the best model for reproducing covariance matrix). When these models fit well the data (i.e., RMSEA is equal or less than 0.05), we can estimate the optimal sample size for fitting the three highly correlated factor model with Teo et al. (2018) pattern matrix or the model with the pessimistic scenario of factor loading matrix fixed to 0.5 and contrasting them with the one-factor model.

All power analyses were performed with powerSem package (Moshagen, 2020) with R software (R Core Team, 2020).

Results of the a priori power computation (Table S1) showed that assuming the Teo et al. (2018) factor loading matrix as the "true population model", the required sample size for fitting a three factor model, with satisfactory fit indices (RMESA = 0.05; SRMR < 0.08; CFI > 0.92; GFI > 0.90; AGFI > 0.90), with a minimum statistical power of 0.90 and alpha level of 0.05, was of N = 101 while assuming a "true population model" with a factor loading matrix equal to that reported by Teo et al. (2018) but with all factor loadings equal to 0.5 (that is a less optimistic model), the required sample for detecting a good fitting model with respect to a one-factor model, was of $N = 399$.

Table S1. Estimates for required sample size, expected fit statistics (RMSEA, SRMS, GFI, AGFI, CFI, normal theory Chi-Square), and power level estimates for finding a good fitting three-factor model against the one-factor model for the two scenarios hypothesized: using Teo et al. (2018) factor loading matrix and the factor loading matrix with factor loadings fixed to 0.5.

	**Teo et al (2018) Factor Loadings, and Latent Correlations of 0.8**	**All factor loadings equal 0.5, and latent correlations of 0.8**
**Statistic**	**Estimates**	**Estimates**
**Required Sample Size**	101	399
**df**	275	275
**Critical Chi-Square**	314.6783	314.6783
**Expected Power (1-beta)** [a]	0.901110	0.900808
**RMSEA**	0.052859	0.026487
**SRMR**	0.036300	0.021667
**Mc**	0.681008	0.908043
**GFI**	0.942091	0.984800
**AGFI**	0.931562	0.982037
**CFI**	0.923644	0.954330

RMSEA: Root Mean Squared Error of Approximation, *SRMR:* Standardized Root Mean Square Residuals, *Mc:* McDonald's transformation of the population minimum of the Maximum-Likelihood fitting function, *GFI:* Goodness of Fit Index, *AGFI:* Adjusted Goodness of Fit Index, *CFI:* Comparative Fit Index.

[a] Alpha level was set equal to 0.05, and the minimum threshold for statistical power was set to 0.90.

References

Moshagen, M. (2020). *semPower: Power Analyses for SEM. R package version 1.1.0.* https://CRAN.R-project.org/package=semPower

R Core Team. (2020). *R: A language and environment for statistical computing. R Foundation for Statistical Computing, Vienna, Austria*. https://www.R-project.org/

Teo, A. R., Chen, J. I., Kubo, H., Katsuki, R., Sato-Kasai, M., Shimokawa, N., Hayakawa, K., Umene-Nakano, W., Aikens, J. E., Kanba, S., & Kato, T. A. (2018b). Development and validation of the 25-item Hikikomori Questionnaire (HQ-25). *Psychiatry and Clinical Neurosciences, 72*(10), 780–788. https://doi.org/10.1111/pcn.12691

Table S2. Descriptive statistics for the HQ-25 as a function of participants who lived alone (n = 48) and participants who did not live alone (n = 324).

	Live Alone			Not live Alone			$t(370)$	Cohen's d	p
	M	SD	Min-Max	M	SD	Min-Max			
**Total HQ-25 score**	21.60	17.04	0–75	23.78	16.75	0–81	0.84	0.13	0.40
**Socialization subscore**	9.06	7.77	0–29	11.28	9.09	0–41	1.61	0.25	0.11
**Isolation subscore**	6.69	6.48	0–24	7.36	6.00	0–31	0.71	0.11	0.47
**Emotional support subscore**	5.85	4.68	0–24	5.14	3.99	0–15	1.14	0.18	0.26

Table S3. Descriptive statistics for the HQ-25 as a function of participants who had no sibling (n = 71) and participants with at least one sibling (n = 301).

	Without Sibling(s)			With Sibling(s)			$t(370)$	Cohen's d	p
	M	SD	Min-Max	M	SD	Min-Max			
**Total HQ-25 score**	25.45	16.87	1–75	23.04	16.75	0–81	1.09	0.14	0.28
**Socialization subscore**	12.28	8.83	0–33	10.69	8.97	0–41	1.35	0.18	0.18
**Isolation subscore**	7.87	5.6	0–24	7.13	6.16	0–31	0.93	0.12	0.35
**Emotional support subscore**	5.29	4.56	0–24	5.21	3.98	0–15	0.15	0.02	0.88
